# Dr. Ward on the Treatment of Asphygia by Flagellation

**Published:** 1842-10-08

**Authors:** 


### ANALECTA.

*Treatment of Asphyxia by Flagellation.*—Dr. T. O. Ward reports the following interesting case in the London Medical Gazette of August 19th. We have seen flagellation very successfully used in rousing the vital powers in cases of poisoning from opium.

On Good Friday, 1840, a very bleak day, I was called into a cottage to see a child that had just been taken out of a mill-stream, in which it had been partly immersed and partly floating for some minutes. I found it cold, insensible, swollen, and moaning at each breath, which was drawn at long intervals. I immediately ordered it to be put into a tub of hot water, before the fire; but the tub was so small, that the women were obliged to pour and dash the warm water over the body while others rubbed it. Finding this of no use, but that the pulse became almost imperceptible, the belly more tumid, and the body colder, I had the child removed from the bath, wrapped in hot blankets, and well rubbed. This produced amendment for a moment only, when seeing a birch rod hanging up, I took a few twigs, and began to whip the child. Instantly the limbs contracted from the pain, the cry was more distinct, the pulse rose, the belly subsided, and warmth was restored.

The frictions were now resumed, and a little hot brandy and water was given, but with slight effect; for the body again became cold, the pulse fell, the abdomen swelled, and life seemed fast ebbing away, when I again had recourse to the scourging, with the same effect as before. Now, in addition to the frictions over the chest, abdomen, and limbs, I applied a mustard plaster to the spine, and gave more brandy and water, but all in vain: I was continually obliged to return to the use of the rod. Presently the parish surgeon came, and we agreed, while waiting for a galvanic machine for which I had sent, to apply boiling water in a bladder to the chest, mustard baths to the feet, and ammonia, which he had brought with him, to the nose. The effect of the boiling water was dreadful to witness. The poor child instantly opened its eyes with a horrid stare, which it continued as long as the application lasted, uttering wailing cries at the same time, and becoming much more roused than before; but when we had removed the hot water, the unfavourable symptoms returned even worse than previously, and we could not recur to this remedy for fear of the consequences. Soon afterwards the galvanic apparatus arrived, and shocks and currents, gradually increased in power, were passed through the chest and diaphragm, but without more permanent effect than any other of the remedies used, except the scourging, which I had continued at intervals all along. I now trusted to this and to the rubbing alone; and in about two hours from my first seeing the child, I had the satisfaction of putting it into bed to its mother (whom I had directed to go to bed, for the purpose of keeping it warm,) with every prospect of its speedy complete recovery.

When I saw it the next morning, I could not recognize in the delicate child before me the turgid features of the day before; and, what is rather remarkable, the only mark of the boiling water was a slight roughness of the cuticle of the chest; nor was the skin marked with weals from the use of the rod. I attribute the long duration of the asphyxia to a bronchial affection under which the child laboured at the time of the accident, but which was little aggravated by it.

The exhalation and reabsorptiou of the intestinal gases, under the diminution and return of nervous energy, in this case, seem to deserve notice, as affording a satisfactory explanation of the source of the tympanitis that attends many diseases affecting the nervous system in general—as hysteria, &c.; or the nerves of the abdomen—as peritonitis and typhus, though in this last disease we may consider the meteorism to have a general as well as a local cause. A late traveller in Norway, whose name I forget, asserts that the Norwegians, in their journeys over the half-frozen rivers, make use of this circumstance to release their horses when they fall through holes in the ice, by throwing a noose over their necks, which they pull tight, till the horse, beginning to be strangled, has its abdomen so much swelled with gas as to float on its back, and is thus easily drawn out of the water over the edge of the ice.

